# 748 Burn Center Family Needs Study Brings Changes to The Unit Practice

**DOI:** 10.1093/jbcr/irac012.301

**Published:** 2022-03-23

**Authors:** Naiwei Hsu, Jasmine Fox

**Affiliations:** Torrance Memorial Medical Center, Torrance, California; Torrance Memorial Medical Center, Torrance, California

## Abstract

**Introduction:**

Pediatric burn patient family needs have many similarities to adult burn patients. However, based on our abstract Family Needs of Adult & Pediatric Burn Patients study from 2013 to 2018, there is a difference. We found our desire to preserve the pediatric burn patient family members as the "safe, comforting person" prevented them from learning all the necessary skills to be the successful caregivers once the child was discharged.

**Methods:**

An integrative review is conducted using a literature search from EBSCOhost, CINAHL, Scopus, Medline, and PubMed. We found very little information addressing this specific concern for our pediatric burn population. However, there is a lot of literature discussing how to involve pediatric family members with other childhood diseases and the benefits of making pediatric family members a part of the treatment team. We shared our literature findings along with the results of responses we got from our Family Needs of Adult & Pediatric Burn Patients study with our Shared Decision-Making (SDM) Council and implemented our new practice.

**Results:**

An informal interview results showed much improved pediatric burn family member satisfaction. By the time the family takes the burn child home, they demonstrate proficient dressing change skills along with the knowledge of how to manage their pain, itch, nutritional needs, and how to contact their care team as needed.

**Conclusions:**

While most of us know the importance of family centered care, putting this concept into practice need more structured approaches and administrative support. Unit based SDM Council and Journal Club can help to keep our nursing staff, especially the new hires to embrace this practice.

